# Enhancing Diagnostic Accuracy of aMCI in the Elderly: Combination of Olfactory Test, Pupillary Response Test, BDNF Plasma Level, and APOE Genotype

**DOI:** 10.1155/2014/912586

**Published:** 2014-02-02

**Authors:** Yuda Turana, Teguh Asaat S. Ranakusuma, Jan Sudir Purba, Nurmiati Amir, Siti Airiza Ahmad, Moh. Hasan Machfoed, Yvonne Suzy Handayani, Asmarinah, Sarwono Waspadji

**Affiliations:** ^1^Department of Neurology, Faculty of Medicine, Atma Jaya Catholic University of Indonesia, Jl. Pluit Raya No. 2, North Jakarta 14440, Indonesia; ^2^Department of Neurology, Faculty of Medicine, University of Indonesia, Jl. Salemba Raya No. 6, Central Jakarta 10430, Indonesia; ^3^Department of Psychiatry, Faculty of Medicine, University of Indonesia, Jl. Salemba Raya No. 6, Central Jakarta 10430, Indonesia; ^4^Department of Neurology, Faculty of Medicine, University of Airlangga, Kampus A, Jl. Mayjen. Prof. Dr. Moestopo No. 47, Surabaya 60131, Indonesia; ^5^Center of Health Research, Atma Jaya Catholic University of Indonesia, Jl. Pluit Raya No. 2, North Jakarta 14440, Indonesia; ^6^Department of Biology, Faculty of Medicine, University of Indonesia, Jl. Salemba Raya No. 6, Central Jakarta 10430, Indonesia; ^7^Department of Internal Medicine, Faculty of Medicine, University of Indonesia, Jl. Salemba Raya No. 6, Central Jakarta 10430, Indonesia

## Abstract

*Background*. Amnestic Mild Cognitive Impairment (aMCI) often progresses to Alzheimer's disease. There are clinical markers and biomarkers to identify the degenerative process in the brain. *Objectives*. To obtain the diagnostic values of olfactory test, pupillary response to tropicamide 0.01%, BDNF plasma level, and APOE **ε**4 in diagnosing aMCI. *Methods*. Cross-sectional, comparative analysis. *Results*. There were 109 subjects enrolled (aMCI: 51, normal cognition: 58) with age 64 ± 5.54 years. For diagnosing aMCI, cut-off point for the olfactory score was <7 out of 10 and >22% for pupil dilatation response. Low BDNF plasma level was related significantly with olfactory deficits and aMCI (*P* < 0.05). Four of five subjects with homozygote e4 presented with multiple-domain aMCI. This group displayed the lowest means of olfactory score and the highest means of pupillary hypersensitivity response (*P* < 0.0001). Combination of olfactory deficit and pupillary hypersensitivity response in detection of aMCI was beneficial with Sp 91% and PPV 87%. In conjunction with clinical markers, BDNF plasma level and presence of APOE e4+ improved Sp and PPV. *Conclusions*. Combination of olfactory test and pupillary response test was useful as diagnostic tool in aMCI. In conjunction with clinical markers, low level of BDNF plasma and presence of APOE e4 improved the diagnostic value.

## 1. Introduction

Mild Cognitive Impairment (MCI) is a transitional state between normal cognitive function and dementia. It is predicted that 50–80% of patients with MCI will eventually develop dementia in the later stage of the disease [1]. The memory-predominant subtype, amnestic MCI (aMCI) conveys the highest risk of progressing to Alzheimer's dementia (AD) [2]. Neuropathologic study also has shown that aMCI seemed to be intermediate between the neurofibrillary changes of aging and the pathologic features of very early AD [3]. Dementia and MCI are clinical diagnosis based on psychometric evaluation. In spite of this evaluation being the gold standard, there are still limitations to its usage (e.g, illiterate patients, visual and hearing problems, and pseudoamnesia). There are clinical and biologic markers available to identify the degenerative process in the brain that have been studied, such as olfactory test [4–16], pupil dilatation response to tropicamide [17–26], APOE genotype [27–34], and brain derived neurotrophic factor (BDNF) plasma level [35–42].

Previous report has featured relationship between several markers with degenerative process and dementia such as olfactory deficits that is now being used as practice parameter in diagnosis of Parkinson disease [43]. Other studies published the presence of olfactory deficit in AD patients [13–16]. Studies performed in aMCI patients also revealed that patients with low olfactory scores were more likely to progress toward dementia [11].

Pupillary hypersensitivity response to tropicamide in dementia patients has been recorded in many studies. Scinto et al. reported a cut-off point of 13% in 30 minutes to differentiate dementia and normal cognition in the elderly using 0.01% tropicamide [17]. Another study published by Iijima et al. using 0.005% tropicamide showed hypersensitivity in pupil dilatation response in AD patients when compared with non-AD subjects [20].

Based on the former reports, olfactory test and pupil dilatation response to tropicamide could be the new potential markers in detecting aMCI. To our knowledge, there has not been a study combining the two factors for diagnostic purpose in aMCI patients. We are also aware of APOE and BDNF as markers of the degenerative process in the brain. Therefore, we want to investigate whether combination of olfactory test and pupillary response to tropicamide 0.01% has diagnosis value and whether presence of APOE *ε*4 and low BDNF plasma level can be useful to enhance diagnostic accuracy of aMCI.

## 2. Methods

This is a cross-sectional study, comparative analysis in elderly with normal cognition and those presenting with aMCI. The study consisted of all subjects participating in the baseline cognitive assessment of study on quality of life in elderly in Kali Anyar, West Jakarta, done by Center of Health Research Atma Jaya Catholic University of Indonesia, about 12 months prior to this study (between August 2011 and September 2011). Inclusion criteria are age ≥60 years old and being literate (reading and writing skills). Exclusion criteria are hearing problems, major psychiatric disorders, depression (*Geriatric Depression Scale*/GDS >4) [44, 45], history of cataract surgery, severe medical illness, past consumption of drugs affecting brain's function and structure, history of cerebrovascular disease, epilepsy, and diabetes mellitus (consumption of antiglycaemic drugs or fasting blood glucose ≥126 mg/dL) [46, 47].

Cognitive assessments were conducted and the results were compared with baseline data. Assessments were done using Forward Digit Span, Clock Drawing Test and MMSE, Verbal Fluency (VF), Boston Naming Test (BNT), Word List Memory Immediate Recall (WLM IR), World List Memory Delayed Recall (WLM DR), Recognition, and Constructional Praxis (CP) from CERAD (Consortium to Establish a Registry for Alzheimer's Disease) Neuropsychological Battery [48].

Diagnosis of MCI was based according to Consortium Criteria proposed by the International Working Group on MCI [49]. The criteria include (1) absence of dementia according to DSM IV or ICD-10, (2) evidence of cognitive decline overtime on objective cognitive task, and (3) preserved baseline activities of daily living or only minimal impairment in complex instrumental functions. Definition of cognitive decline is decrease ≥2 points/year in one cognitive instrument (MMSE/WLM IR/WLM DR) or decrease ≥1 point/year in at least 2 cognitive instruments (MMSE/WLM IR/WLM DR). Single-domain aMCI was diagnosed if subjects are showing deficit in memory task (Saving Score <65% or WLM IR (third repetition) <8), but not in any other area of cognitive domains. Multiple-domain aMCI was diagnosed if there was presence of memory deficit and also in other cognitive domains. Nonamnestic MCI (naMCI) was diagnosed if there was impairment in cognitive domains other than memory (we excluded naMCI in this study). The regional ethical committee approved the study and written informed consent was obtained from each individual.

### 2.1. Apolipoprotein E and BDNF Plasma Level

Blood samples (10 mL) were collected from each subject. Routine blood test was performed along with fasting blood glucose, lipid profile, APOE, and BDNF plasma level. APOE and BDNF were done blinded for all clinical data. APOE measurement was completed using Restriction Fragment Length Polymorphism (RFLP) method. In this study, we used High Pure Polymerase Chain Reaction (PCR) Template Preparation Kit for extraction and PCR was performed using FastStart Taq DNA Polymerase (Roche Applied Biosystem). BDNF was measured using ELISA kit from R&D Systems.

### 2.2. Olfactory Test

Examination of olfactory nerve function was performed using 10 odors commonly found in Indonesia: cajuput oil, coffee, jasmine, menthol, tobacco, kerosene, *pandan*, camphor, chocolate, and orange [50]. The odors were preserved in similar containers, sealed, and coded continuously. Subjects were allowed to smell the odors twice for 5 seconds before being asked to identify them. They were given a 30 sec break prior to identifying the next odor.

### 2.3. Pupillary Response to 0.01% Tropicamide

The examiner instilled a drop of 0.01% tropicamide on one subject's eye, while the other eye received saline as control. We measured pupil diameter at 30 minutes, 40 minutes, and 50 minutes [17] using Colvard pupillometry in a semidarkened room [51, 52]. Pupillary response was examined using Granholm et al. method by measuring anisocoria (percent difference between tropicamide and saline eyes at each time point). This method was preferred to reduce bias resulting from fatigue, stress, drugs, and others that might influence the pupil size [53].

### 2.4. Statistical Analysis

Differences in proportions were assessed by means of chi-square, Mann-Whitney tests, or Kruskall-Wallis tests. The tests were performed to compare the demographic and clinical factors between subjects with aMCI and cognitively normal individuals in both groups. Any significant items were then entered into a multivariate logistic regression to develop a model for predicting aMCI, using stepwise selection with an inclusion criteria of *P* < 0.05. Statistical analysis was performed using the SPSS 15.0 software (SPSS Inc., Chicago, IL, USA). The level of significance was set at *P* < 0.05 for all statistical analyses.

## 3. Results

**Figure 1:**
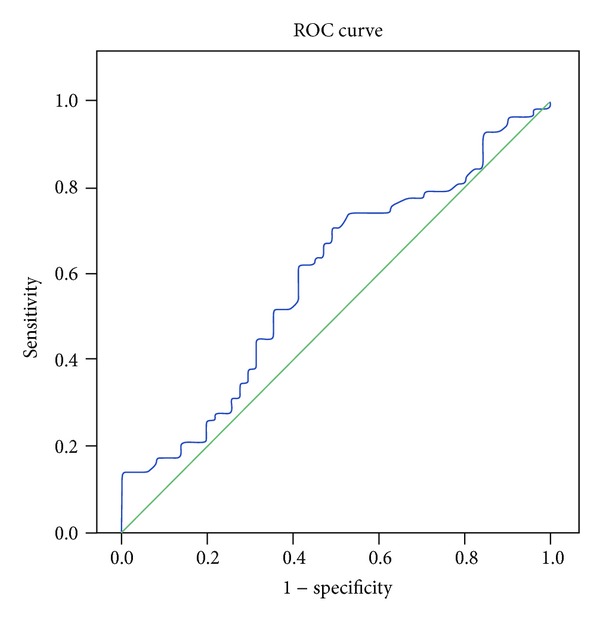
The ROC curve of BDNF plasma level and aMCI.

**Figure 2:**
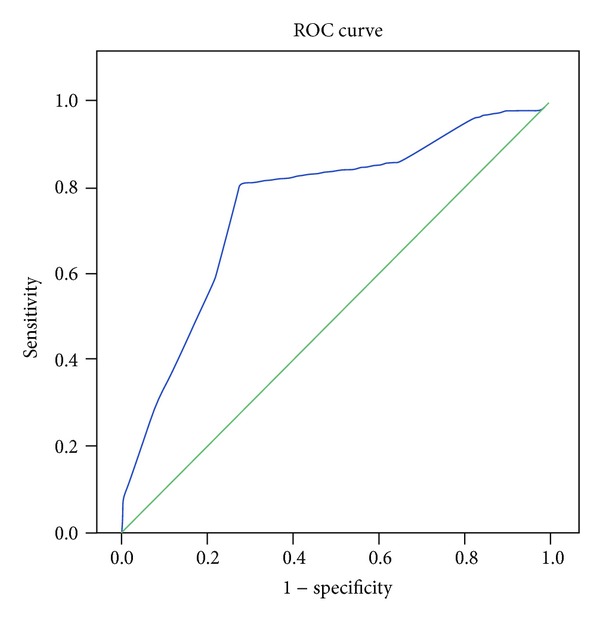
The ROC curve of olfactory deficit and aMCI.

**Figure 3:**
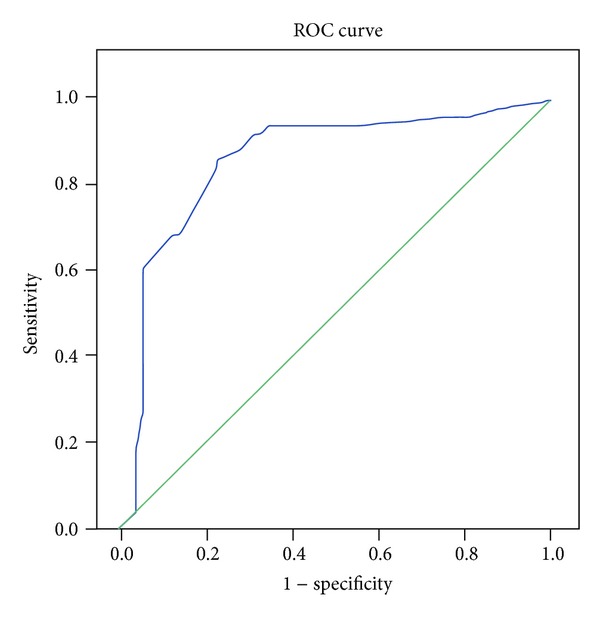
The ROC curve of pupillary response and aMCI.

There were 109 subjects enrolled in this study (normal cognition: 58, single-domain aMCI: 10, and multiple-domain aMCI: 41); 77 subjects were women (70.6%) and most of the subjects had <6 years of formal education (40%) (Table 1). Using ROC curve, we calculated the cut-off points to determine aMCI as follows: cut-off point for low BDNF plasma level ≤1314 pg/mL, less than 7 for olfactory deficit, and pupillary hypersensitivity response >22% (maximal peak diameter on 30 minutes) (Figures 1, 2, and 3).

The majority of subjects had multiple-domain aMCI (35%). There was no significant relationship between aMCI and APOE *ε*4 genotype. However, four of five subjects with *ε*4 homozygote also showed multiple-domain aMCI. Group with multiple-domain aMCI had the lowest means of olfactory score and the highest means of pupillary hypersensitivity response (*P* < 0.0001) (Table 2).

After performing chi-square test, we identified three variables with *P* < 0.05: pupillary response to tropicamide, olfactory nerve deficit, and BDNF plasma level (Table 1). We further analyzed using logistic regression test and found significant relationship between pupillary hypersensitivity response (OR = 13.69) and olfactory nerve deficit (OR = 5.99) with aMCI.

There was no significant relationship between *ε*4 genotype with olfactory scores and pupillary response (*P* > 0.05). However, we observed that subjects with *ε*4/*ε*4 genotypes scored the lowest in olfactory test and had the highest pupillary response. There was also significant relationship between low BDNF plasma level with lower olfactory scores (*P* = 0.012).

Combination of olfactory nerve deficit and pupillary hypersensitivity response generated good values of specificity/Sp 91%, positive predictive value/PPV 87%, and negative predictive value/NPV 75% when differentiating aMCI from those with normal cognition. In conjunction with clinical markers, low BDNF plasma level and presence of *ε*4+ substantially increased specificity and PPV (Table 3).

In the end of the study, we replicated the test on different group (30 subjects) and the results were almost identical. Combination of olfactory deficit and pupillary hypersensitivity response in differentiating aMCI from normal cognitive function yielded Sv 70%, Sp 95%, PPV 88%, and NPV 86%.

## 4. Discussion

The majority of the subjects in this study came from low educational background. This finding is similar to other study of the elderly population in Indonesia [44]. This may also explain the high prevalence of aMCI (43%) found in our study. The prevalence is higher compared to a study by Luck et al. that stated that the prevalence of aMCI was 17% among elderly aged >65 years old [54].

Four of five subjects with *ε*4+/+ also showed multiple-domain aMCI. Blom et al. reported that seven of eight aMCI patients with *ε*4+/+ genotypes eventually progressed to dementia [55]. We found significant difference in pupillary response and olfactory scores for each type of MCI. Pupil dilatation response in those with multiple-domain aMCI was higher in comparison with normal cognition (*P* < 0.0001). Arai et al. stated that elderly with AD demonstrated pupil dilatation up to 43% when compared to 15.6% in those with normal cognition [56]. In this study, multiple-domain aMCI subjects scored the lowest olfactory score compared to those with single-domain aMCI and normal cognition (Table 3). It has been widely known that multiple-domain aMCI has the worst outcome and a predisposing factor toward dementia [57].

Higuchi et al. reported a significant relationship between *ε*4+ and pupil hypersensitivity response [21]. In a study by Wang et al., they demonstrated the difference between subjects with and without *ε*4 allele in identifying the odors [9]. In our study, subjects with *ε*4 homozygote significantly demonstrated the lowest olfactory scores and highest pupillary hypersensitivity response.

We did not find a significant relationship between APOE and aMCI. Due to the multifactorial nature, APOE solely could not be held responsible for the disease. Despite the fact that APOE *ε*4 genotype is a risk factor that accelerates degenerative process in the brain (AD), *ε*4 carrier status alone could not be accountable for cognitive decline or dementia [58].

The peak dilatation response to 0.01% tropicamide was about 30 min in this study which was similar to other reports [17, 25, 56, 59, 60]. Pupillary response to tropicamide was previously studied to expose degenerative process in Edinger Westphal nucleus. This area demonstrated degeneration in early stage of AD [61]. Pupillary response is an objective test that does not require neuropsychological examination/interview. This can be beneficial in special cases where patients are not able to participate in neuropsychological test. However, this test is not suitable for those with eye disorders such as history of cataract surgery and in diabetes mellitus patients (in whom autonomy nervous system has been compromised).

In our study, the cut-off point for pupil dilatation is >22% for differentiating elderly with aMCI. There are various cut-off points in other publications that may due be to different patients criteria, different age group, and concentration of tropicamide being administered. Scinto et al. reported a cut-off point of 13% in 30 minutes to differentiate dementia and normal cognition in the elderly using 0.01% tropicamide [17]. Another report by Iijima et al. used a cut-off point of 14.5% in 60 minutes using less concentrated tropicamide (0.005%) [20].

In our study, we established the cut-off point <7 for olfactory score to diagnose aMCI in elderly. Eibenstein et al. set a higher cut-off point ≥10 (out of 12) to differentiate elderly with normal cognition and aMCI and stated that scores ≤6 were anosmic [7]. A study by Tabert et al. using 10 different odors set a cut-off point ≤7 in differentiating DA with aMCI [8]. Olfactory test is not similar with cognitive assessment because the former is not influenced by educational level nor depression; hence, it is suitable to use in elderly patients with low level of education and depression [4].

Lower level of BDNF was observed in aMCI group than elderly with normal cognition (*P* = 0.04). This is supported by a study by Lee et al., where they observed low level of BDNF in MCI and dementia patients [40].

High LDL level is undoubtedly a risk factor for vascular disease but its connection with cognitive dysfunction has not yet been established. Yasuno et al. declared no relationship between level of LDL, triglycerides, and total cholesterol with cognitive scores [62]. Another study by Elias et al. also showed no relationship between aMCI and cholesterol, specifically between total cholesterol and memory domain [63]. Reitz et al. stated that plasma lipid levels in the elderly are not associated with the risk of MCI [64]. The relationship between HDL and cognitive function is still inconclusive. Van Exel et al. stated that low HDL level was related to low MMSE scores and these low scores of MMSE did not result from the atherosclerosis process [65]. In contrary, Gillum and Obisesan did not find any significant relationship between HDL level and cognitive function [66].

There was no significant relationship between systolic and diastolic hypertension with aMCI in our study. The issue of hypertension and aMCI and dementia remains controversial. Vascular disease is a risk factor for developing MCI in some studies [67, 68]. Farmer et al. stated that there was no relationship between hypertension and cognitive dysfunction [69]. In another research conducted in Jakarta involving 1001 elderly patients with mean age 68 ± 7 years old, there was no relationship between recall memory and total MMSE scores with hypertension [70]. A different finding is shown by Reitz et al. using 918 subjects, followed for mean of 4.7 years where they found hypertension as a risk factor for nonamnestic MCI (naMCI) not for aMCI [71].

In this study, 13 of 14 subjects with olfactory deficit and *ε*4+ genotypes presented with aMCI (OR = 20). Graves et al. stated that subjects with olfactory deficit and *ε*4+ genotype were 4.9 times likely to suffer from cognitive decline compared to those without olfactory deficit and *ε*4– [10]. We also observed subjects with pupillary hypersensitivity and having *ε*4+ genotype was 4.7 times likely to have aMCI. Hence, presence of *ε*4+ genotype may substantially increase specificity and positive predictive value toward aMCI. In addition, our study showed that 22 out of 26 patients with olfactory deficit and low BDNF plasma level had aMCI (OR = 10).

Based on the findings in our study, biological markers, such as APOE and BDNF, when used in conjunction with clinical markers of pupillary response or olfactory test can increase positive predictive value (PPV) toward aMCI diagnosis. Our findings supported the statement by Lautenschlager et al. that combination of biological and clinical markers is essential to increase PPV in MCI diagnosis [72].

Multivariate analysis in our study revealed that olfactory deficit and pupillary response were related significantly to aMCI. We also found that combination of olfactory test and pupillary response to tropicamide was the best model when considering Sp and PPV (Sv 64,7%, Sp 91,4%, and PPV 86,8%) in comparison to the other combination. We do acknowledge the limitation of our study that we did not perform analysis for combinations of three or more variables due to sample size. To our knowledge, this is the first research combining the two variables at one time. Taking into consideration that the two markers can aid the psychometric evaluation for diagnosis (not for screening purpose), hence, higher specificity is more important than sensitivity.

When we replicated the test on different group (30 subjects), the results for diagnostic values of olfactory deficit and pupillary response were almost identical. The combination of two clinical markers can increase the specificity up to 95% and PPV up to 88%. This shows that the diagnostic values of pupillary response to tropicamide and olfactory deficit in diagnosing aMCI were consistent.

We share optimism that, in the future, combination of these two clinical markers (olfactory test and pupillary response to tropicamide) can be widely implemented together with cognitive assessment. Hence, clinicians can perform early diagnosis of the degenerative process in the brain using various alternatives and institute proper treatment for a better quality of life in the elderly.

## Figures and Tables

**Table 1:** Demographic and clinical status in elderly with aMCI and normal cognition.

Variable	aMCI	Normal	*P*	Odds ratio	95% CI
Age (years)					
(a) ≤65	36 (45.6%)	43 (54.4%)	0.84	0.84	0.36–1.94
(b) >65	15 (50.0%)	15 (50.0%)			
Gender					
(a) Men	16 (50.0%)	16 (50.0%)	0.82	1.20	0.53–2.74
(b) Women	35 (45.5%)	42 (54.5%)			
Years of education					
(a) <6 years	20 (45.5%)	24 (54.5%)	0.97	0.91	0.42–1.97
(b) ≥6 years	31 (47.7%)	34 (52.3%)			
Body mass index (BMI)					
(a) Overweight (≥25.0 kg/m^2^)	26 (48.1%)	28 (51.9%)	0.93	1.11	0.53–2.37
(b) Normal (<25.0 kg/m^2^)	25 (45.5%)	30 (54.5%)			
APOE genotype					
(a) *ε*4+	14 (46.7%)	16 (53.3%)	1.00	1.00	0.43–2.32
(b) *ε*4−	36 (46.8%)	41 (53.2%)			
BDNF level					
(a) Low (≤1314 pg/mL)	26 (60.5%)	17 (39.5%)	0.04	2.51	1.14–5.52
(b) High (>1314 pg/mL)	25 (37.9%)	41 (62.1%)			
Olfactory deficit					
(a) Yes (Skor 0–6)	37 (77.1%)	11 (22.9%)	<0.0001	11.29	4.59–27.76
(b) No (Skor 7–10)	14 (23.0%)	47 (77.0%)			
Pupillary hypersensitivity to tropicamide					
(a) Yes (>22%)	44 (77.2%)	13 (22.8%)	<0.0001	21.76	7.94–59.65
(b) No (≤22%)	7 (13.5%)	45 (86.5%)			
Systolic BP					
(a) Hypertension (≥140 mmHg)	23 (44.2%)	29 (55.8%)	0.75	0.82	0.39–1.75
(b) Normal (<140 mmHg)	28 (49.1%)	29 (50.9%)			
Diastolic BP					
(a) Hypertension (≥90 mmHg)	29 (52.7%)	26 (47.3%)	0.29	1.62	0.76–3.46
(b) Normal (<90 mmHg)	22 (40.7%)	32 (59.3%)			
LDL					
(a) High (≥130 mg/dL)	35 (52.2%)	32 (47.8%)	0.21	1.78	0.81–3.90
(b) Normal (<130 mg/dL)	16 (38.1%)	26 (61.9%)			
HDL					
(a) Low (<40 mg/dL for men, <50 mg/dL for women)	18 (46.2%)	21 (53.8%)	1.00	0.96	0.44–2.11
(b) Normal (≥40 mg/dL for men, ≥50 mg/dL for women)	33 (47.1%)	37 (52.9%)			

**Table 2:** Pupillary response and olfactory score in elderly with aMCI and normal cognition.

Variable	aMCI		Normal	P*
	Single domain	Multiple domain		
Pupillary response (%)	30.4 ± 9.30	36.06 ± 15.85	17.20 ± 13.81	<0.0001
Olfactory score	6.10 ± 1.60	5.80 ± 1.89	7.53 ± 1.68	<0.0001

*Kruskal-Wallis test.

**Table 3:** Combination of olfactory deficit, pupillary hypersensitivity response, APOE *ε*4, and BDNF plasma level in elderly with aMCI and normal cognition.

	aMCI	Normal	Sv (%)	Sp (%)	PPV (%)	NPV (%)	OR (95% CI)	*P*
Olfactory deficit								
(a) Yes	37 (77.1%)	11 (22.9%)	72.6	81.0	77.1	77.1	11.29 (4.59–27.76)	<0.0001
(b) No	14 (23.0%)	47 (77.0%)						
Pupillary hypersensitivity response								
(a) Yes	44 (77.2%)	13 (22.8%)	86.3	77.6	77.2	86.5	21.76 (7.94–59.65)	<0.0001
(b) No	7 (13.5%)	45 (86.5%)						
Olfactory deficit and APOE *ε*4*								
(a) Yes	13 (92.9%)	1 (7.1%)	26.0	98.3	92.9	60.2	19.68 (2.47–156.85)	0.01
(b) No	37 (39.8%)	56 (60.2%)						
Olfactory deficit and low BDNF plasma level								
(a) Yes	22 (84.6%)	4 (15.4%)	43.1	93.1	84.6	65.1	10.24 (3.22–32.57)	<0.0001
(b) No	29 (34.9%)	54 (65.1%)						
Pupillary hypersensitivity response and APOE *ε*4*								
(a) Yes	13 (76.5%)	4 (23.5%)	26.0	92.9	76.5	58.9	4.66 (1.41–15.41)	0.016
(b) No	37 (41.1%)	53 (58.9%)						
Pupillary hypersensitivity response and low BDNF plasma level								
(a) Yes	20 (79.9%)	6 (23.1%)	39.2	89.7	76.9	62.7	5.59 (2.03–15.43)	0.001
(b) No	31 (37.3%)	52 (62.7%)						
Olfactory deficit and pupillary hypersensitivity response								
(a) Yes	33 (86.8%)	5 (13.2%)	64.7	91.4	86.8	74.7	19.43 (6.59–57.34)	<0.0001
(b) No	18 (25.4%)	53 (74.6%)						

Note. *APOE *ε*2/*ε*4 genotype is not included.
